# Shall Medicines Be Administered before, with or after Eating?

**Published:** 1887-06

**Authors:** J. Edward Green

**Affiliations:** Macon, Ga., Secretary Macon Medical Society


### SHALL MEDICINES BE ADMINISTERED BEFORE, WITH OR AFTER EATING?

---

BY J. EDWARD GREEN, M. D., MACON, GA., SECRETARY MACON MEDICAL SOCIETY.

---

All medicines being poisons to a certain extent, and setting up an abnormal action in the human organism, I think, whenever practicable, it is best to exhibit them upon a full stomach. Of course there are a great many exceptions to be made to the above statement, but there are two grand divisions to which it especially applies, and those are remedies which are principally constructive in their action and those which act as irritants. It will readily be seen that the latter class are rendered much more acceptable to the patient by being mixed with his food than after fasting; when coming in immediate contact with the branches of the pneumogastric nerve, it sets up a violent and convulsive action of the whole alimentary canal in order to expel the offending material, thus frequently eliminating the substance from the body before it has remained a sufficient length of time to have had its due effect on the system. In no case is this better illustrated than in the administration of opium. Give a dose of that drug to a person unaccustomed to its use, and what is the result? In a great many cases, violent nausea, headache and vertigo are immediately set up, the patient has a pale, anxious face, the pulse decreases in fullness, the brow is overspread with a cold, clammy sweat, the bowels are constipated, and the only secreting function in the whole body not interfered with is the perspiration. It is of course desirable in all medication to produce the one effect required, and no more, but failing in this, in order to relieve a neuralgic pain at the tip end of the finger or some remote part of the body, it becomes necessary to bring the whole organism under the effect of the drug, and bring about such a violent and abnormal action in the system as above described, but by directing it to be taken after eating, all its disagreeable effects are greatly diminished, much to the patient's comfort and satisfaction, and the one effect desired on the nervous system, to soothe pain and promote sleep, is much better attained. How well is this shown in some conditions of asthenia and irritability where alcohol is administered. Taken into the viscus in an empty condition, oxidation proceeds at a greatly increased rate, the heart pumps twice as fast as natural, all the functions are stimulated to increased exertion, and a large amount of muscular and nerve tissue is actually burned up, leaving the patient in a more exhausted and worse condition than before. If taken with or after food the effect is almost reversed. Instead of a rapid and transient excitement, the patient is sustained and strengthened, elimination goes on a moderate speed and a universal sense of *bien etre* is secured. Its calmative effects when exhibited in this way are sometimes wonderful. It will hardly be disputed that all tonics had best be taken on a full stomach, for given when fasting, the organ is immediately stimulated to pour out its natural secretion, when, having nothing to act upon, it is either re-absorbed or else does a positive injury by it corrosive influence upon the mucous membrane. Tonics, given to induce an artificial appetite, rarely succeed unless the above rule be observed.

Even the seemingly small consideration of disguising the disagreeable taste of drugs is best accomplished by taking them with food, and this is by no means so unimportant a matter as is generally supposed, for we frequently hear of a patient, ladies especially, discharging their medical adviser because, as they say, "his medicine was so bad to take." In this respect the homœopathists have the advantage of us in the elegance and nicety of their preparations. Of course a little humbuggery is pardonable in everything, and some trifling wrinkle of administering castor oil or other black draughts has been known to insure a practitioner's success. Cod liver oil is another instance. Some patients, to whom it is best given in a pure state, find it absolutely impossible to retain it on an empty stomach, yet absorb and assimilate it with perfect ease when mixed with their nourishment. The reasons for this are two-fold: 1st, ingested when the organ is empty, it irritates the pneumogastric nerve, which promptly causes its expulsion; 2nd, the gastric juice exerts little or no solvent power on pure oily matters, and consequently the effect is "nil." However, when thoroughly incorporated with the matters taken into the body, it offers no hindrance to the natural action of the stomach's secretion.

To quote another instance: The books all tell us to give arsenic after a full meal, whether used as a tonic or anti-periodic. Arsenic is not only an irritant, but has a tendency to accumulate in the liver and other parts of the body. These effects are in a great degree obviated when carried through the circulation with the pabulum. Another reason against administering drugs while the stomach is contracted is that the gastric juice works a more complete chemical change coming in direct contact with the substance than when it is mixed with other material, thus oftentimes so modifying its chemical properties as to render it partially or wholly inert. It is well known that morphia takes longer to act and more is required when taken in the ordinary way than when introduced hypodermically, owing to the decomposition it undergoes in the process of absorption.

One more point: In order to affect a certain organ or locality, it is of course necessary that the medicine shall permeate the whole current of the circulation. This cannot be accomplished, as before explained, when such a degree of irritation is set up that the substance is immediately rushed out of the system by the spontaneous action of the stomach and bowels and before it can have been absorbed. In cachectic subjects—for example, in the latter stages of syphilis, when it is necessary to feed the patient on large doses of the iodide of potassium—the irritation set up by its administration between the hours of meals would more than counterbalance the good effects otherwise to be expected from its use; also in the tubercular dyscrasia, when the salts of iron are used with much success to avert the accession of phthisis, when not taken in conjunction with the nutriment, its effect is little beyond that of an astringent. This agent in particular, in order to secure its full therapeutical effects, requires to become part of the food itself during the digestive process, thereby being thoroughly assimilated and absorbed and carried through every portion of the economy, performs its proper function and builds up the system broken down by overwork, malnutrition and disease. If Jacob's idea is correct, that chlorate of potassium destroys the mucous coats of the stomach and intestines, this deleterious effect is certainly lessened, if not entirely obviated, by giving it in the way advocated. This also applies to the bromides and other salts of potash when it is necessary, as in some cases of epilepsy, to feed the patient on large quantities. In certian cases of dyspepsia, where acids are indicated, owing to a deficiency of the stomach's natural secretion, it seems useless to administer it dry so, but rather let it be taken while the digestive process is in progress, when it may act as an artificial digestor until the organ resumes its natural tone; otherwise it is immediately absorbed, or else carried far down in the intestinal tract where it can affect nothing.

And so we might go on through the whole pharmacopæia. To sum up, then, it may be broadly stated that all medicines which are tonics are irritant in their action, all constructive agents, all alteratives, all remedies which are to be thoroughly carried into the system, and *par excellence* all alcoholic stimulants had best be given with or immediately after a meal.

